# Exploring new horizons in neuroscience disease detection through innovative visual signal analysis

**DOI:** 10.1038/s41598-024-54416-y

**Published:** 2024-02-20

**Authors:** Nisreen Said Amer, Samir Brahim Belhaouari

**Affiliations:** Division of Information and Computing Technology, College of Science and Engineering, Hamad Bin Khalifa University, 34110 Doha, Qatar

**Keywords:** Neuroscience, Diseases, Medical research

## Abstract

Brain disorders pose a substantial global health challenge, persisting as a leading cause of mortality worldwide. Electroencephalogram (EEG) analysis is crucial for diagnosing brain disorders, but it can be challenging for medical practitioners to interpret complex EEG signals and make accurate diagnoses. To address this, our study focuses on visualizing complex EEG signals in a format easily understandable by medical professionals and deep learning algorithms. We propose a novel time–frequency (TF) transform called the Forward–Backward Fourier transform (FBFT) and utilize convolutional neural networks (CNNs) to extract meaningful features from TF images and classify brain disorders. We introduce the concept of eye-naked classification, which integrates domain-specific knowledge and clinical expertise into the classification process. Our study demonstrates the effectiveness of the FBFT method, achieving impressive accuracies across multiple brain disorders using CNN-based classification. Specifically, we achieve accuracies of 99.82% for epilepsy, 95.91% for Alzheimer’s disease (AD), 85.1% for murmur, and 100% for mental stress using CNN-based classification. Furthermore, in the context of naked-eye classification, we achieve accuracies of 78.6%, 71.9%, 82.7%, and 91.0% for epilepsy, AD, murmur, and mental stress, respectively. Additionally, we incorporate a mean correlation coefficient (mCC) based channel selection method to enhance the accuracy of our classification further. By combining these innovative approaches, our study enhances the visualization of EEG signals, providing medical professionals with a deeper understanding of TF medical images. This research has the potential to bridge the gap between image classification and visual medical interpretation, leading to better disease detection and improved patient care in the field of neuroscience.

## Introduction

**Figure 1:**
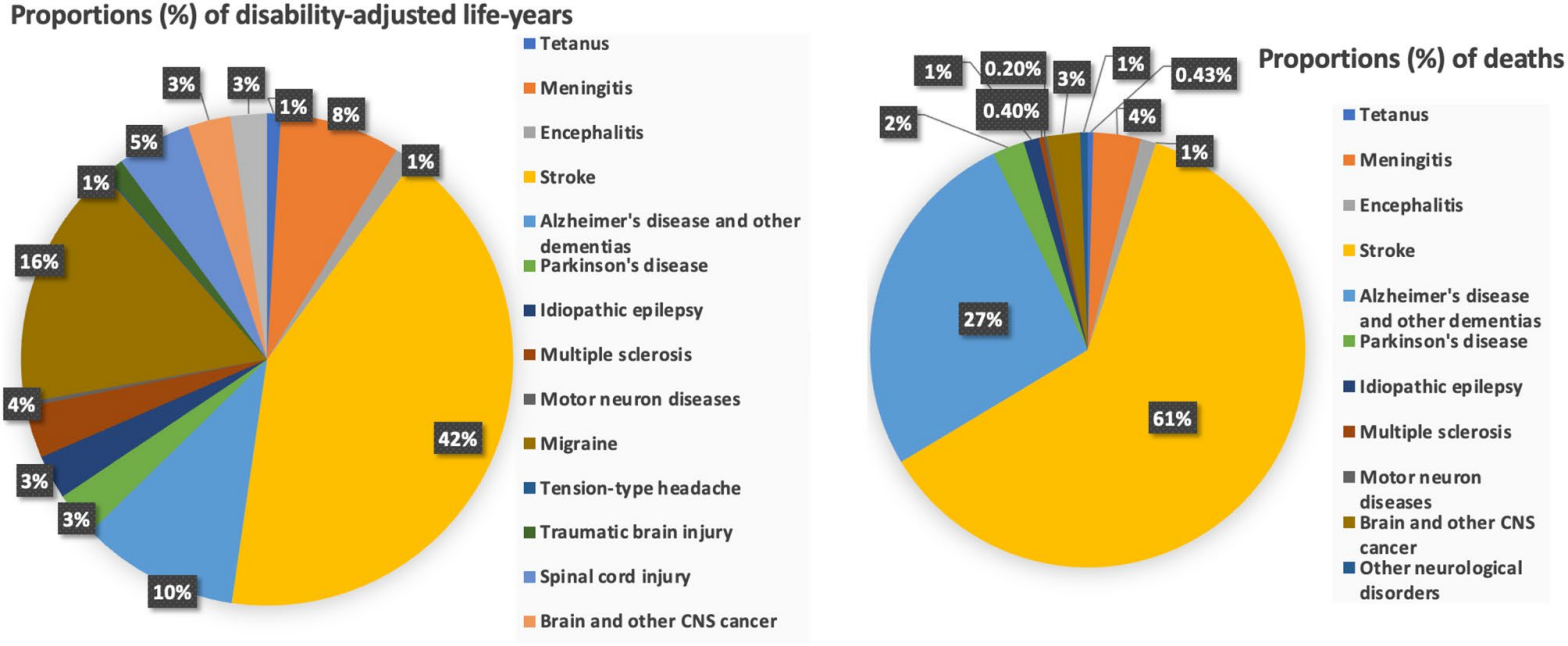
Proportional contribution of various brain disorders to the overall burden of neurological disorders: (**a**) Proportions (%) of disability-adjusted life-years and (**b**) deaths.

Brain disorders are a growing global health concern, particularly in low- and middle-income countries. Deaths and disabilities from brain disorders have surged by 39% and 15% in the last 3 decades, making them the second leading cause of global mortality, resulting in approximately 9 million deaths annually, as shown in Fig. 1. Access to neurological disorder services and support remains inadequate, especially in less affluent nations.

In response to this alarming trend, the World Health Organization (WHO) endorsed an action plan at the 75th World Health Assembly in May 2022. This plan focuses on improving diagnosis, treatment, care, research, and innovation while strengthening information systems for brain disorders. Our research aligns with the WHO’s strategic plan, aiming to automatically and accurately diagnose brain disorders using a novel technique. Electroencephalogram (EEG) signals provide valuable information about brain health but suffer from low amplitude, high noise, and limited interpretability^1^. While ML/DL models show promise in diagnosing brain diseases from EEG, they face challenges with limited datasets, inter-subject variability^2^, and generalization^3^. Many studies have explored automatic diagnosis of brain disorders from EEG signals, such as stroke in elders^4^, Parkinson’s disease (PD)^5^, Alzheimer’s^6^, autism^7^, and ADHD^8^. However, most focus on event-related potentials (ERPs) or Fourier-based power analyses, which have limitations in capturing the full spectrum of EEG data^9^.

To address these challenges, our research employs time–frequency (TF) transformations to enhance the visualization and interpretation of EEG data. This improved representation enables accurate classification using machine learning (ML) and deep learning (DL) models, aiding pathologists and neurologists.

Our research focuses on transforming complex EEG signals into interpretable representations, bridging the gap between DL models and medical professionals’ expertise. This empowers doctors to make more informed decisions based on visual data. This combined approach of visual interpretation and DL-based classification yields improved results.

Key contributions of our work include:

- Channel selection method based on mean correlation coefficients (mCC): We propose a channel selection method that identifies key EEG channels for brain disorder classification using mCC. This method helps improve the accuracy of classification.
- Transformation of EEG signals into 2D images using the Forward–Backward Fourier transform (FBFT): We enhance the interpretation of EEG signals by transforming them into 2D images using the novel FBFT technique. We compare this technique with other time–frequency transforms to demonstrate its effectiveness.
- Concatenation of time–frequency images from selected channels: We propose a method to concatenate time–frequency images from selected channels into a single input for ML/DL models. This approach improves the classification accuracy of brain disorders.
- Analysis and comparison of pre-trained DL models: We analyze and compare pre-trained DL models for diagnosing brain disorders using FBFT-transformed EEG images. This analysis helps identify the most effective models for accurate classification.
- Naked-eye diagnosis approach: We propose a naked-eye diagnosis approach for brain disorders based on time–frequency images obtained through FBFT. This approach integrates visual interpretation with DL-based classification, improving diagnostic accuracy.

Several deep learning methods have been proposed for brain disorder’s classification^10–12^, but none of these methods have analyzed the models on different time–frequency transformed images of EEG. Additionally, most of these methods focus on diagnosing a single brain disorder from EEG Signals.

In this paper, we revolutionize EEG visual interpretation and provide novel time–frequency images for deep learning models. These images enhance diagnostic accuracy and foster effective collaboration between humans and machines, advancing medical imaging and diagnosis significantly. Our proposed models outperform the state-of-the-art in disease diagnosis.

## Related work

In the realm of epileptic seizure classification, various innovative methodologies have been proposed, each offering distinct contributions to the field.

The authors of^13^ introduced a novel approach for epileptic seizure classification, utilizing Discrete Fourier Transform (DFT) and an Attention Network AttVGGNet. The method achieved an accuracy of 95.6% and other notable performance metrics. Similarly, the authors of^14^ improved epilepsy diagnosis accuracy using EEG recordings by combining DFT with brain connectivity measures and feeding the data into an Autoencoder Neural Network. The approach achieved an accuracy of 97.91%, sensitivity (SENS) of 97.65%, and specificity (SPEC) of 98.06%. In^15^, spectrogram and scalogram images from Short Time Fourier Transform (STFT) were employed for ictal-preictal-interictal classification using a Convolutional Neural Network (CNN), achieving 97% accuracy. Researchers like^16^ and^17^ also used CNNs for EEG classification from STFT, achieving accuracies of 91.71% and 97.75%, respectively. Utilizing Discrete Wavelet Transform, the authors of^18^ achieved an accuracy of 95.6% in ictal-interictal EEG signal classification. In^19^, an adaptive approach with Pattern Wavelet Transform and a Fuzzy classifier achieved ACC of 96.02% and SPEC of 94.5% for ictal-interictal classification. The authors of^20^ used a hybrid of DWT and LDA classifier, resulting in an accuracy of 99.6% and SENS of 99.8% for the ictal-interictal problem. In^21^, the authors proposed a multiscale short-time Fourier transform for feature extraction coupled with a 3D convolutional neural network. The approach demonstrated accurate seizure detection with a 14.84% rectified predictive ictal probability error and a 2.3s detection latency.

Several studies have shown promising outcomes in identifying neurological disorders like Alzheimer’s disease (AD) to enhance the quality of life for affected individuals. The authors of^22^ implemented a method using techniques for extracting distinctive attributes and categorizing EEG, achieving accuracies differentiating between AD patients, those with mild AD, and individuals in a healthy group. They utilized scalograms generated from Fourier and Wavelet Transforms, achieving accuracies of 83% for AD versus normal cases, 92% for healthy versus mild AD cases, and 79% for mild versus AD classification scenarios. The authors of^23^ used time-dependent power spectrum descriptors for CNN input, achieving an accuracy of 82.30% in a dataset of 64 AD, 64 MCI, and 64 HC subjects. Similarly, the authors of^24^ collected resting-state EEG signals from individuals with mild cognitive impairment (MCI), AD, and healthy controls (HC). They used functional connectivity measures from EEG data as input for a convolutional neural network (CNN), achieving recognition accuracy rates of 93.42% for MCI and 98.54% for AD.

Commencing with stress detection, notable studies have employed advanced techniques to identify and classify emotional states based on EEG data accurately. The authors of^25^ explored stress detection using the DEAP dataset, employing power spectrum-based feature extraction on all 32 channels with 5-second windows. The AlexNet architecture was utilized for classification, distinguishing between calm and distressed emotional states with an accuracy of 84%. The authors of^26^ conducted stress detection using EEGMAT data, applying the Discrete Wavelet Transform (DWT) to 19 out of 23 EEG channels. The classification, facilitated by a Convolutional Neural Network-Bidirectional Long Short-Term Memory (CNN-BLSTM) architecture, successfully differentiated between stressed and relaxed states, achieving an impressive accuracy of 99.2%. Similarly, the authors of^27^ employed the SEED dataset, combining filtering and a music model for feature extraction across all 62 EEG channels. Using an Artificial Neural Network (ANN), they classified emotions into neutral, positive, and negative categories, achieving an impressive accuracy of 97%. Another noteworthy study in^28^ utilized the DASPS dataset, employing power spectrum-based feature extraction on all 14 EEG channels. The classification, performed in 1-second windows with a total duration of 15 seconds, utilized K-Nearest Neighbors (KNN) to distinguish between binary and four-class anxiety levels, achieving an accuracy of 83.8%.

For the heart murmur, author^29^ conducted a study employing feature extraction through Fast Fourier Transform (FFT) on 942 cases with 6 EEG channels. The dataset was segmented into 4-second intervals with a 1-second overlap. The authors applied a combination of deep learning models, specifically DBResNet and XGBoost, achieving accuracies of 76.2% and 82%, respectively. Subsequently, the authors of^30^ utilized a convolutional neural network (CNN) for feature extraction. This study reported an enhanced classification accuracy of 87.2%. The authors of^31^ extended the investigation, focusing on an unspecified feature extraction method for the 942 cases. The study adopted an all-inclusive approach by considering all EEG channels and applying a CNN for classification, yielding an accuracy of 75.7%.

## Methodology

**Figure 2:**
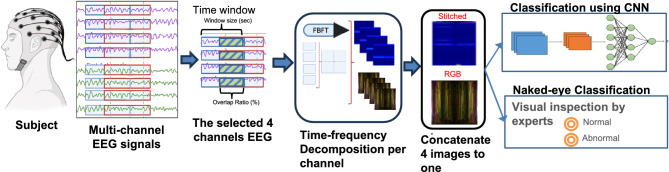
Flowchart of the proposed model for brain disorders classification from EEG signals.

Our approach encompasses multiple steps, including EEG dataset collection for various brain disorders, signal pre-processing, TF analysis for 2D image generation, deep learning (DL), and human visual classification, as illustrated in Fig. 2. We’ve implemented this approach in Python, utilizing pre-trained neural network models on TF images from brain disorders such as epilepsy, Alzheimer’s, murmur, and stress EEG. Model evaluation employs established performance metrics.

### Data description and pre-processing

EEG signals of subjects with various brain disorders, including epilepsy, Alzheimer’s, murmur, and stress, are analyzed in this study. For the diagnosis of epilepsy, the CHBMIT dataset developed in Boston Children’s Hospital is used^32^. 23 channels EEG sampled at 256 samples/s are recorded from 22 subjects, 5 males and 17 females, aged between 1.5 and 22 years old. For stress, we utilized the dataset by Bird et al.^33^, recorded using a Muse headband with four dry EEG sensors (TP9, AF7, AF8, and TP10). It covers three mental states: relaxed, neutral, and concentrating, comprising 25 recordings from five participants, each with two-minute sessions for each mental state.

For diagnosing Alzheimer’s disease (AD), we utilized the Open-Neuro dataset, comprising EEG data from 28 participants at the Department of Neurology, AHEPA General University Hospital of Thessaloniki, Greece. These participants were categorized into three groups: Alzheimer’s disease patients (AD), frontotemporal dementia patients (FTD), and a control group (CN) consisting of healthy age-matched adults^34^. The EEG signals were sampled at 500 Hz with a resolution of 10 V/mm, and the duration of EEG recordings varied across groups.

The study also covers heart murmurs, utilizing the MIT Physionet dataset^35^. This dataset comprises heart sound recordings collected during screening campaigns in Northeast Brazil in 2014 and 2015^36^. It includes recordings for 1568 participants, ranging from 5 to 45 seconds in length, resulting in 5272 recordings. The recordings are categorized by the valve’s location (PV, AV, MV, TV, or other), and each participant is labeled for the presence, absence, or unknown status of heart murmurs.

### EEG channels selection using Pearson’s correlation coefficient

Channel selection is crucial to effectively diagnose brain disorders from multi-channel EEG datasets. While using all channels is an option, it often results in redundancy, increased feature count, computational complexity, and memory demands^37^. Hence, selecting a subset of channels is a recommended practice. This selection can be performed visually by a neurophysiologist or through an automated algorithm. In our approach, we propose a correlation-based method. Initially, the correlation coefficient between two EEG channels, denoted as $x_1$ and $x_2$, is computed as follows:

1 $$\begin{aligned} |r_{x_1 x_2}| = \left| \frac{\sum _{i=1}^{n} (x_{i1} - {\overline{x}}_1)(x_{i2} - {\overline{x}}_2)}{\sqrt{\sum _{i=1}^{n} (x_{i1} - {\overline{x}}_1)^2 \sum _{i=1}^{n}(x_{i2} - {\overline{x}}_2)^2}}\right| \end{aligned}$$

where *n* is the total number of samples, $\overline{x_1}$ and $\overline{x_2}$ are the mean and $x_{i1}$, $x_{i2}$ are the *ith* samples of the two channels. This correlation coefficient is computed for each channel with all other channels, resulting in a correlation matrix (CorrMat) given as follows:

2 $$\begin{aligned} CorrMat = \begin{bmatrix} r_{x_1 x_1} &{} r_{x_1 x_2} &{} \ldots &{} r_{x_1 x_n} \\ r_{x_2 x_1} &{} r_{x_2 x_2} &{} \ldots &{} r_{x_2 x_n} \\ \vdots &{} \vdots &{} \ddots &{} \vdots \\ r_{x_n x_1} &{} r_{x_n x_2} &{} \ldots &{} r_{x_n x_n} \end{bmatrix} \end{aligned}$$

**Figure 3:**
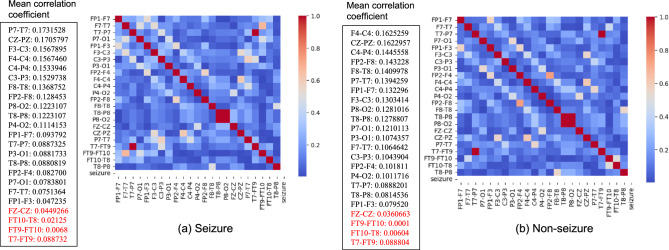
Correlation Matrix for two EEG from (**a**) Seizure, (**b**) non-seizure subjects and the mean correlation values of each channel.

The mean correlation coefficient for each channel is calculated by computing the mean of each column in the CorrMat. Examples of the CorrMat and mean values for two randomly selected subjects, one with seizures and one without, are shown in Fig. 3. Channels with low mean correlation coefficients are selected and further validated by neurophysiologists. Highly correlated channels may capture redundant information, increasing noise and reducing classification accuracy. In contrast, uncorrelated channels provide unique information, improving accuracy. For the two subjects in Fig. 3, the channels with the lowest mean correlation coefficients are identified as FZ-CZ, FT9-FT10, FT10-T8, T7-FT9, and are selected for further feature extraction.

### Time–frequency (TF) analysis to transform the EEG signals to 2D images

Time–frequency analysis (TF) of EEG signals offers advantages over both time and frequency domain analyses^38^. It tracks changes in brain wave amplitude and phase across time and frequencies, enhancing interpretability by measuring fundamental brain properties. TF analysis transforms signals into informative 2D time–frequency images, revealing variations among brain disorders, easily classifiable using deep CNN models. Previous studies have focused on a single TF method to create 2D scalogram images^39,40^. Common TF techniques include Short-time Fourier Transform (STFT)^41^, continuous wavelet transform (CWT)^42^, discrete wavelet transform (DWT)^43^, Hilbert transform (HT)^44^, and empirical mode decomposition (EMD)^45^.

Each TF method has its pros and cons. For instance, STFT is computationally efficient but can produce blurred TF representations due to windowing. Wavelet transforms offer precision but can be computationally demanding. The Hilbert transform is relatively straightforward but noise-sensitive, while EMD is robust but more complex to implement and interpret.

To address these limitations, we introduce the novel FBFT transform, providing superior 2D TF image visualization, detailed in the next subsection.

#### The FBFT transform

The Forward–Backward Fourier Transform (FBFT) process is a sophisticated signal processing technique employed to extract critical time information from Electroencephalogram (EEG) signals in the domain of brain activity analysis. This multifaceted method encompasses several essential steps. Initially, the EEG signal is partitioned into subarrays for streamlined processing. Subsequently, zero padding is applied to these subarrays, extending the signal’s length and enhancing the frequency analysis’s precision. The Fast Fourier Transform (FFT) is then executed on these zero-padded subarrays, facilitating the conversion of the signal from the time domain to the frequency domain for detailed analysis of its frequency components. The FBFT procedure involves an in-depth analysis of the minimum magnitude values resulting from both the forward and backward transformations, aiding in identifying dominant frequency components in the EEG signal. This comprehensive methodology allows for extracting essential spectral and time-varying features from EEG signals by eliminating signal frequency harmonies. This process provides valuable insights for disease diagnosis and analysis in neuroscience research. The FBFT process, which encapsulates the Forward–Backward Fourier Transform, can be mathematically represented by the following equation:

3 $$\begin{aligned} \begin{aligned} X(f, u) = \min \Biggl \{\int _{-\infty }^{+\infty } e^{-j 2 \pi f t} x(t) 1_{\{t<u\}} d t, \int _{-\infty }^{+\infty } e^{-j 2 \pi f t} x(t) 1_{\{t>u\}} d t\Biggr \} \end{aligned} \end{aligned}$$

Where *X*(*f*, *u*) represents the result of the operation or transformation for a given frequency *f* and time *u*, *f* represents the signal frequency, *u* represents the time variable, *e* is the mathematical constant that represents the base of the natural logarithm, *j* is an imaginary unit, *x*(*t*) is the input signal as a function of time *t*, $1_{\{t<u\}}$ is an indicator function that equals 1 if *t* belongs to the interval *u* and is zero otherwise. The mathematical foundation is further elucidated below.

*Fourier transform* The Fourier Transform is a powerful tool in signal processing and mathematics used to convert a signal from its original time or space domain into the frequency domain. The equation for the Fourier Transform is:

4 $$\begin{aligned} X(f)=\int _{-\infty }^{+\infty } x(t) e^{-2 \pi j f t} d t \end{aligned}$$

*Inverse Fourier transform* The Inverse Fourier Transform converts the frequency-domain signal back into its original time-domain form. This process is crucial for understanding how a signal can be reconstructed from its frequency components. It is given by the equation:

5 $$\begin{aligned} x(t)=\int _{-\infty }^{+\infty } X(f) e^{+2 \pi j f t} d t \end{aligned}$$

*Unit function* This function is fundamental in signal processing for representing binary states or switches.

6 $$\begin{aligned} U(t)=\left\{ \begin{array}{l}1 \geqslant 0 \\ 0<0\end{array}\right. \end{aligned}$$

*Fourier transform of unit function*

7 $$\begin{aligned} U(f)=\int _{-\infty }^{+\infty } u(t) e^{-2 \pi f t} d t=\frac{1}{2}\left( \delta (f)+\frac{1}{j \pi f}\right) \end{aligned}$$

Where

8 $$\begin{aligned} \delta (f)=\left\{ \begin{array}{cc} +\infty &{} f=0 \\ 0 &{} f \ne 0 \end{array} \quad \& \int _{-\varepsilon }^{+\varepsilon } \delta (f) d f=1, \forall \varepsilon >0\right. \end{aligned}$$

$\delta (f)$ is called Dirac Delta function and is crucial in sampling and reconstructing signals. It is infinitely high at $f=0$ and zero elsewhere, with the integral over a small region around zero equal to 1.

*Time-shifted Fourier transform* For a time-shifted signal, if Fourier transform of *x*(*t*) is *x*(*f*)

9 $$\begin{aligned} \begin{aligned} \Rightarrow F T\left( x\left( t-t_0\right) \right)&=\int _{-\infty }^{+\infty } x\left( t-t_0\right) e^{-2 \pi j t f} d t \\ \end{aligned} \end{aligned}$$

If we put:

10 $$\begin{aligned} \begin{aligned}t-t_0=t^{\prime }&\Rightarrow F T\left( x\left( t-t_0\right) \right) =e^{-2 \pi j f t_0} X(f) \end{aligned} \end{aligned}$$

Fourier transform of $1-U\left( t-t_0\right)$ :

11 $$\begin{aligned} \begin{aligned}{}&{\text {FT}}[1-U(t-t_0)]=F T(1)-F T(U(t-t_0)) \\&\quad =\delta (f)-e^{-2 \pi j t_0 f} F T(U(t)) \\&\quad =\delta (f)-\frac{e^{-2 \pi j t_0f}}{2}\left[ \delta (f)+\frac{1}{j \pi f}\right] \\&\quad =\delta (f)-\frac{e^{-2 \pi j t_0f}}{2j \pi f}-\frac{1}{2} \delta (f) e^{-2 \pi j t_0 f} \\&\quad =\delta (f)-\frac{e^{-2 \pi j t_0f}}{2 j \pi f}-\frac{1}{2} \delta (f) \\&\quad =\frac{1}{2} \delta (f)-\frac{e^{-2 \pi j t_0f}}{2 \pi j f} \\&\quad =\frac{1}{2}\left[ \delta (f)-\frac{e^{-2 \pi j t_0f}}{j \pi f}\right] \end{aligned} \end{aligned}$$

It results in a combination of the delta function and a phase-shifted inverse frequency term.

**Figure 4:**
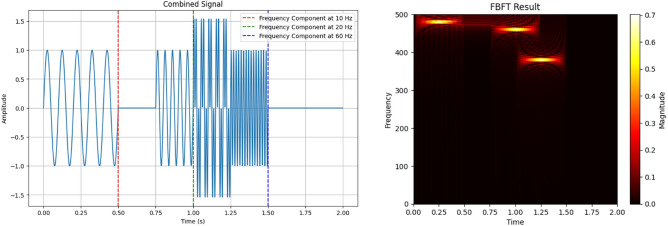
FBFT on a combined signal with frequencies 10, 20, and 60.

Figure 4 visually demonstrates FBFT’s effectiveness in EEG signal analysis, offering insights into both time and frequency domains. It showcases FBFT’s ability to provide a comprehensive representation of a combined signal with frequencies of 10, 20, and 60, aiding in the understanding of the signal’s time-varying behavior and frequency component distribution.

**Figure 5:**
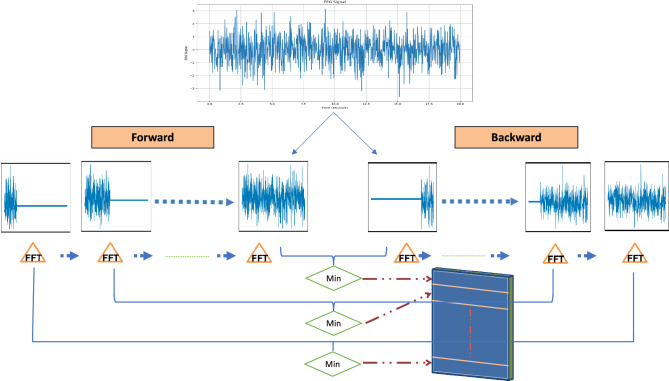
Using FBFT to analyze the signal.

Figure 5 demonstrates FBFT for forward and backward signal analysis applied to a simulated EEG signal. The accompanying pseudocode in Table 1 outlines the FBFT process for practical implementation.

**Figure 6:**
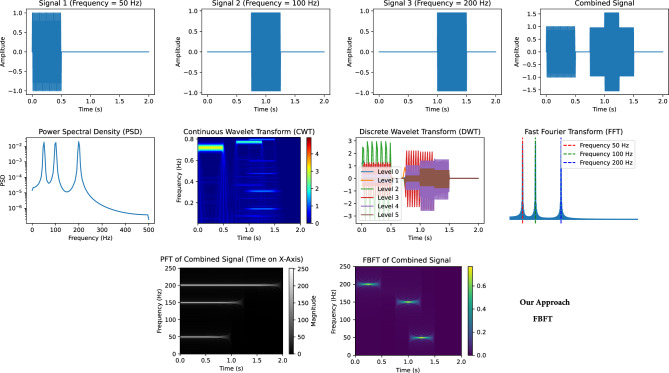
A synthetic composite signal with three oscillations of different frequencies combined at different timings and its corresponding plots in time domain (i.e. (t vs x(t) top row), frequency domain (FFT, PSD) and TF CWT and DWT in the middle row, the PFT and the proposed TF plot using FBFT in the bottom row.

Figure 6 provides a comparative analysis between FBFT, traditional signal processing techniques such as FFT^41^, Continuous Wavelet Transform (CWT)^42^, Discrete Wavelet Transform (DWT)^43^, the Power Spectrum (PS)^46^, and the Progressive Fourier Transform (PFT)^47^ when applied to a composite signal with known frequency components. FBFT distinguishes itself by its impressive capability to achieve precise time–frequency localization. In contrast, while FFT provides frequency information, it lacks the precision to pinpoint these frequency components in the time domain precisely. The Progressive Fourier Transform (PFT) technique enables the extraction of time–frequency-related insights from signals, but it doesn’t provide the same level of precision as FBFT when pinpointing and accurately characterizing frequency components within the time domain. Similarly, although CWT and DWT offer improved time–frequency resolution compared to FFT, they may still have limitations in capturing subtle shifts in the signal. The Power Spectrum (PS) represents another valuable analysis tool, illustrating the frequency content of the signal, but it may not provide the same time–frequency precision as FBFT. This illustrates the distinct advantages of FBFT in EEG signal analysis, combining the benefits of both time and frequency domain analysis, allowing for precise localization of frequency components.

**Table 1:** Pseudocode FBFT.

Step	Description
1	Initialize an empty list M to store intermediate results.
2	Iterate over each index u in the range of the length of the signal array.
3	Slice the signal array from the beginning up to index u+1 and assign it to H.
4	Apply the Fast Fourier Transform (FFT) to H.
5	Append the result to the M list.
6	Initialize an empty list M1 to store reverse iteration results.
7	Iterate over each index u in the range of the length of the signal array.
8	Slice the signal array from -u-1 to the end and assign it to H1.
9	Apply the FFT to H1.
10	Append the result to the M1 list.
11	Compute the element-wise minimum between the reversed M1 and M lists.
12	Return the resulting array as min_values.

### CNN-based classification of brain disorders

Deep learning, particularly CNNs, excels in pattern recognition and image classification, automatically extracting features from raw input images^48^. CNNs, including AlexNet^49^, GoogLeNet^50^, and SqueezeNet^51^, were employed for brain disorder diagnosis. These models have fixed input sizes: 256 $\times$ 256 $\times$ 3 for AlexNet, 224 $\times$ 224 $\times$ 3 for GoogleNet, and 227 $\times$ 227 $\times$ 3 for SqueezeNet. Input images were resized using cubic interpolation for each dataset. Random augmentations, such as flipping, translating, and scaling, were applied during training. Key parameters included the ADAM optimization algorithm, a mini-batch size of 16, an initial learning rate of 0.0001, and a maximum of 50 epochs. Model performance was assessed using metrics like accuracy, F1 score, sensitivity, recall, and precision, with an 80–20% train/test split.

### Naked-eye classification of brain disorders using TF images of EEG

Our study primarily focuses on visually classifying brain diseases using transformed EEG images, a vital component to enhancing disease understanding and detection through visual and deep learning techniques. As part of our study, we conducted a brief 4-5 minute survey. Volunteers assess reference images of brain diseases (e.g., epilepsy, Alzheimer’s) and normal ones without disease indicators. Participants are initially tasked with visually studying these labeled images to identify distinct patterns or distinguishing features corresponding to each label. Subsequently, they are asked to provide their own judgment regarding whether an image appears normal or abnormal. Throughout this survey, we strictly adhere to ethical standards, encompassing informed consent, data privacy, voluntary participation, and institutional ethical approval. Our survey aims to collect valuable input and insights from participants, significantly contributing to the development of practical approaches for healthcare practitioners in the visual analysis-based detection of brain and heart diseases. The experimental protocols utilized in this study were approved by Hamad Bin Khalifa University Institutional Review Board (HBKU-IRB) under the reference number ’HBKU-IRB-2024-68’. The HBKU-IRB, as the named institutional review committee, conducted a comprehensive review in line with ethical standards and regulatory requirements. Participants provided informed consent, and the study was conducted in adherence to the approved protocols and principles covered in the CITI program.

## Results

The main goal of this research is to improve the interpretability of EEG signals by using the novel FBFT transform to convert them into time–frequency (TF) images. This transformation enhances the accuracy of visual inspection and deep learning-based classification of brain diseases. By representing brain activity as sinusoidal oscillations instead of voltage changes at specific time points, we gain a better understanding of EEG signals. These sinusoidal wave-like patterns capture brain oscillations, and TF analysis provides insights into their frequency, amplitude, and phase over time.

For illustration, this study generates two types of TF images from the signals. The RGB and concatenating images to generate scalo- grams or spectrograms for each time-framed window of EEG data, as illustrated in Fig. 2. Table 2 shows an example of TF analysis and its corresponding 2D TF images for the stress dataset. Furthermore, Table 3 displays the FBFT images for the four datasets used in this study.

**Table 2:**
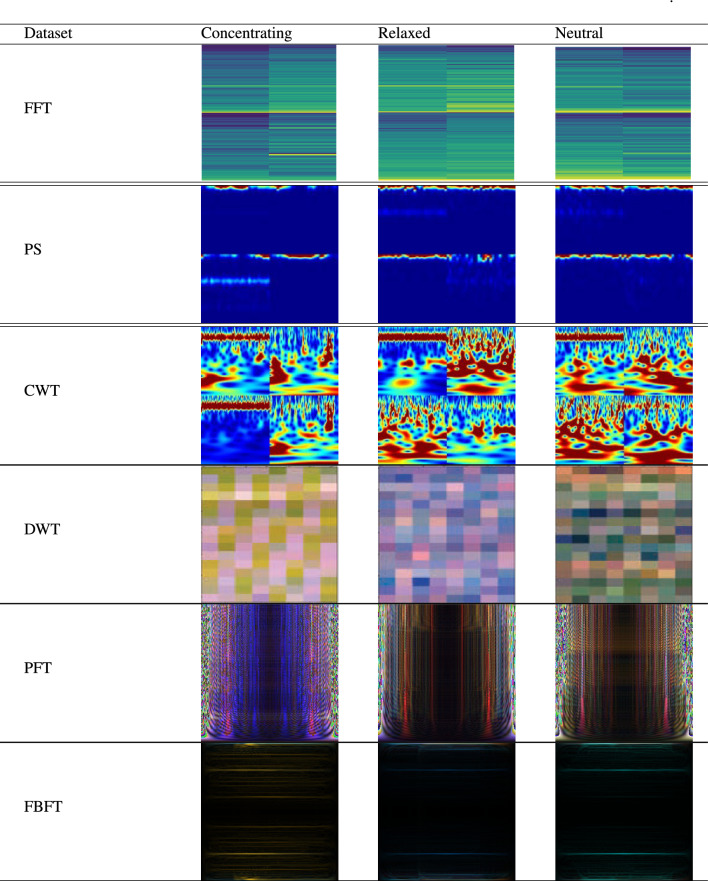
Sample images of TF transforms applied to EEG from the stress dataset^33^.

**Table 3:**
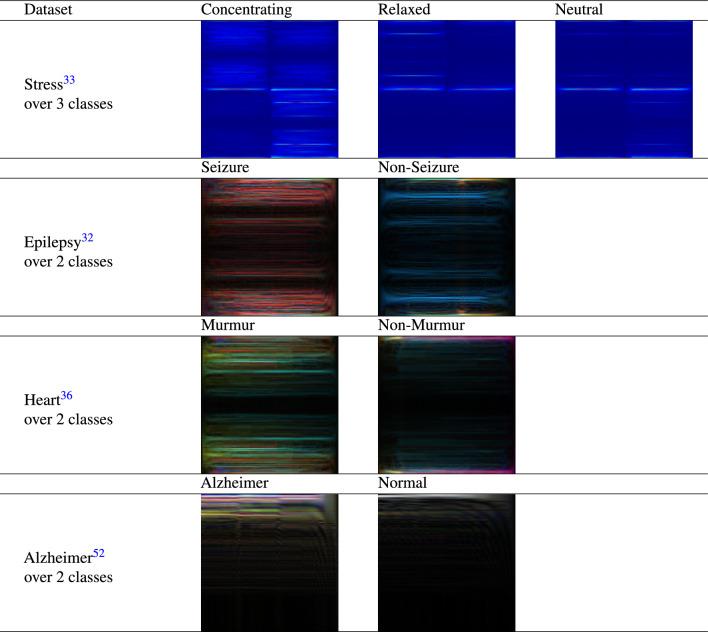
Sample images for different datasets generated using Forward Backward Fourier Transform (FBFT).

**Figure 7:**
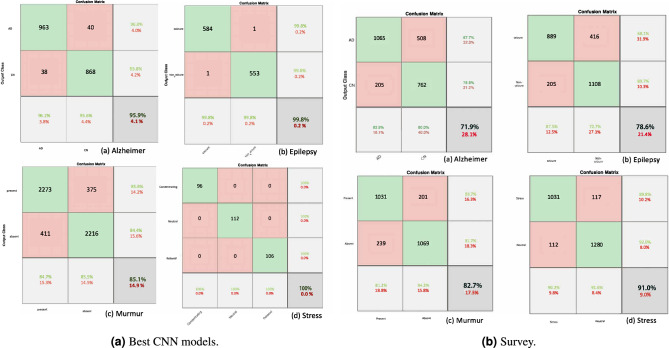
Confusion matrices for: (**a**) best CNN models and (**b**) Manual visual classification.

In parallel with these visual representations, three pre-trained models—GoogleNet, SqueezeNet, and AlexNet— were employed to diagnose various brain disorders. Performance metrics are summarized in Table 4, and Fig. 7a shows the confusion matrices for the best models. For epileptic seizure diagnosis^32^, the four selected channels ’T7-FT9/FT9-FT10/FT10-T8/’FZ-CZ’ were converted to TF RGB images using the proposed FBFT transform with a 1-second time window and 0.25-second overlap. The accuracies achieved by the three models were 99.56%, 99.82%, and 99.39%, respectively. For Alzheimer’s disease diagnosis^52^, six optimal channels P3/O2/T6/O1/F8/Pz were selected, and the FBFT with a 2-second time window and 1.5-second overlap was applied to obtain RGB TF images. The models achieved accuracies of 95.91%, 93.03%, and 91.72%, respectively. In the context of murmur detection, the MIT Physionet dataset is used, which contains heart sound recordings^35^. Following the removal of NaN values, the FBFT transform was applied to the selected four channels ’PV, TV, MV, and AV’, resulting in TF images for both murmur and no murmur classes. The models achieved accuracies of 85.1%, 81.56%, and 79.64%, respectively. For automatic detection of stress^33^, four channels ’TP9, AF7, AF8, and TP10’ were transformed to TF images using the proposed FBFT transform, with a 1-second time window and no overlap. The three pre-trained models achieved accuracies of 100%, 99.36%, and 99.36%, respectively.

Another significant contribution of this research involved the visual classification of brain disorders by medical experts without relying on automated algorithms. A survey with 125 participants was conducted. They visually classified disorders by examining novel FBFT images of EEG signals related to the four datasets. The survey included the classification of 10,000 images, resulting in overall accuracies of 78.6%, 71.9%, 82.7%, and 91.0% for epilepsy, Alzheimer’s disease (AD), murmurs, and mental stress, respectively. Detailed classification outcomes are presented in the confusion matrix in Fig. 7b.

**Table 4:** Performance Metrics for FBFT-based CNN Models.

Dataset	CNN	Acc.	Prec.	Sen.	F1 score
Stress^33^	GoogleNet	100	1	1	1
			1	1	1
			1	1	1
Murmur^36^	GoogleNet	85.1	0.8584	0.8469	0.8526
			0.8435	0.8553	0.8494
ALZ^52^	GoogleNet	95.91	0.9601	0.9620	0.9611
			0.9581	0.9559	0.9570
CHBMIT^32^	SqueezeNet	99.82	0.9983	0.9983	0.9983
			0.9982	0.9982	0.9982

## Discussion

This study introduces FBFT, a novel method for transforming EEG signals into time–frequency (TF) images, enhancing their visual interpretability. These TF images are then used to train pre-trained CNN models for diagnosing epilepsy, Alzheimer’s disease, murmurs, and mental stress. Additionally, we explore manual classification by visually inspecting TF images, demonstrating the effectiveness of our models. Table 5 compares our model’s performance with existing methods for diagnosing these disorders. Our pre-trained models, utilizing FBFT-based TF images from just four to six EEG channels, consistently outperform alternatives in accuracy. This study also introduces a manual classification approach using TF images, showcasing its effectiveness. Expanding our approach to diverse neurological disorders presents challenges, including adapting to inherent EEG signal variability and securing representative datasets. The necessity for diverse datasets for each disorder may pose challenges in data availability. Considerations for generalization involve addressing individual variations in brain activity and potential use of disorder-specific feature extraction techniques. Computational demands of the FBFT and CNN models may increase, requiring optimizations for scalability. Addressing these challenges involves thoroughly understanding each disorder’s unique characteristics and continuous methodology refinement. While promising for specific disorders, applying the method to a broader range requires addressing challenges related to variability, dataset diversity, and computational efficiency. These considerations offer a nuanced view of the method’s applicability, signaling areas for future research and development.

The initial survey, involving 125 participants, has yielded promising accuracy, serving as a foundation for future research endeavors. Subsequent investigations will encompass more extensive participant cohorts and heightened statistical analyses. Although our current methodology demonstrates proficiency in classifying four specific disorders, its applicability extends to a broader spectrum of neurological conditions using EEG data. However, we acknowledge the necessity for validation in such expansions. Recognizing the critical nature of visual classification by medical professionals, our study diligently addresses the associated reliability and potential biases. We openly acknowledge the constraints of subjective interpretation, emphasizing the importance of inter-rater reliability assessments, and recognizing the impact of individual expertise on classification accuracy. To foster transparency and mitigate biases, we have expanded our discussion on ethical considerations, elucidating survey details such as participant numbers, duration, and specific instructions. Furthermore, we advocate for the continued enhancement of our manual classification approach by integrating additional diagnostic criteria and comparative analyses with automated algorithms. Detailed insights into classification outcomes, presented in Fig. 7b, offer valuable perspectives on observed patterns in the confusion matrix. Our outlined future research plans underscore a commitment to larger participant pools, more robust statistical analyses, and potential extensions to diverse neurological conditions, collectively contributing to a nuanced comprehension of the human interpretation and validation processes intrinsic to our study.

**Table 5:** Comparison of the proposed models with SOTA for the diagnosis of epileptic seizure, AD, murmur, and stress.

Year	Author	DataSet	Features extraction	No. of cases	No. of EEG channels	EEG segmentation	Classifier	Accuracy (%)
2023	^53^	Epilepsy^32^	FFT	22	22	1s/0.5s	SVM	97.7
2023	^54^	Epilepsy^32^	Sparse CSP	7	19	1s/no	ASTF	98.81
2023	^55^	Epilepsy^32^	WT + PS	22	18	2s/no	CNN	94.5
2022	^56^	Epilepsy^32^	EMD	22	22	10s/no	MLPNN	99.57
2023	^57^	Epilepsy^32^	STN	22	20	4s/2s	KNN, RF	97.81
2022	^58^	Epilepsy^32^	DWT	23	22	30s/1s	SVM	96.38
Our method		Epilepsy^32^	FBFT	22	4	1s/0.25s	GoogleNet AlexNet SqueezeNet	99.56 99.39 **99.82**
2020	^59^	Stress^33^	SF	5	4	–	MLP	97.18
2021	^60^	Stress^33^	SF	5	4	–	SVM	91.6
2021	^61^	Stress^33^	SF	5	4		RF	96.69
Our method		Stress^33^	FBFT	5	4	1s/no	GoogleNet AlexNet SqueezeNet	**100** 99.36 99.36
2022	^29^	Murmur^36^	FFT	942	6	4s/1s	DBRes ResNet, XGBoost	76.2 82
2022	^30^	Murmur^36^		942			CNN	87.2
2023	^31^	Murmur^36^	NA	942	All	-	CNN	75.7
Our method		Murmur^36^	FBFT	140	4	1s/no	GoogleNet AlexNet SqueezeNet	**85.1** 79.64 81.65
2021	^52^	AD^52^	SF	AD(10) FTD(10) CN(8)	19	5s/2.5s	RF (FTD/CN)	86.3
2023	^62^	AD^52^	SVD	AD(10) FTD(10) CN(8)	19	4s/2s	DT (AD/CN) LGBM (AD/CN)	78.5 79.64
2023	^63^	AD^52^	DWT	AD(36) FTD(23) CN (29)	19	30s/15s	FTD/CN Novel DICE	82.67 83.28
Our method		AD^52^	FBFT	AD(10) CN(8)	6	2s/1.5s	GoogleNet AD/CN AlexNet AD/CN SqueezeNet AD/CN	**95.91** 93.03 91.72

Significant values are in (bold).

## Conclusion

In summary, this study presents the Forward Backward Fourier Transform (FBFT), a groundbreaking method for transforming EEG signals into interpretable time–frequency (TF) images, showcasing its effectiveness in terms of accuracy and computational efficiency, particularly when applied to a limited number of EEG channels. The proposed FBFT time–frequency transform provides a robust means of converting EEG data into 2D images, a critical step in analyzing dynamic signals like EEG. Leveraging these TF images, we employed various pre-trained CNN models to diagnose brain disorders, achieving remarkable accuracy rates as detailed in the results section. Notably, our approach maintains computational efficiency by focusing on a selected set of EEG channels, rendering it practical for real-world applications.

A noteworthy innovation in this research is the introduction of naked-eye-based classification, where human experts visually assessed TF images. This manual evaluation achieved noteworthy accuracy rates of 78.6%, 71.9%, 82.7%, and 91.0% for epilepsy, AD, murmur, and mental stress, respectively, presented in Fig. 7b. This visual inspection underscores our method’s potential as a complementary tool for brain disorder diagnosis. Beyond its immediate applications, this methodology holds promise for developing advanced EEG-based diagnostic tools for various neurological disorders. Our approach is robust, adaptable, and extensible, offering the potential to expand the scope of neurological disorders studied and to create real-time applications that assist specialists in the efficient and automated identification of various neurological conditions from EEG data. However, several limitations should be considered. Firstly, the study focused on specific brain disorders, such as epilepsy, Alzheimer’s disease (AD), murmur, and mental stress, leaving room for exploration of its applicability to a more comprehensive spectrum of neurological conditions. Additionally, the reliance on pre-trained CNN models, while effective, introduces a dependency on the quality and applicability of these models to diverse datasets. It is crucial to acknowledge that the accuracy rates achieved, mainly through naked-eye-based classification, may vary based on the expertise of human assessors and the inherent subjectivity of visual inspection.

In conclusion, this study introduces the Forward Backward Fourier Transform (FBFT) as a groundbreaking method for transforming EEG signals into interpretable time–frequency (TF) images. The research showcases its effectiveness, particularly in accuracy and computational efficiency, mainly when applied to a limited number of EEG channels. The proposed FBFT time–frequency transform serves as a robust means of converting EEG data into 2D images, a critical step in analyzing dynamic signals like EEG. Leveraging these TF images, our study employed various pre-trained CNN models, achieving remarkable accuracy rates, as detailed in the results section. An essential advantage of our approach is its computational efficiency, emphasizing a selected set of EEG channels, making it practical for real-world applications. This efficiency ensures quicker and more accessible diagnosis, enhancing its potential for widespread clinical use and making it a valuable tool in the realm of neurological disorder detection and diagnosis.

As a future direction, we plan to conduct extensive testing across diverse datasets, exploring alternative neural network architectures beyond pre-trained CNN models. Additionally, we aim to develop user-friendly interfaces for real-time applications, streamlining the efficient and automated identification of neurological conditions by healthcare specialists. Collaborative efforts with experts in neurology and related fields will be instrumental in refining and validating the methodology across a broad spectrum of neurological disorders. Moreover, we anticipate conducting longitudinal studies and clinical trials to thoroughly assess the robustness and reliability of our approach in real-world medical scenarios.

## Data Availability

The datasets analyzed during the current study are available in specified repositories. The Murmur Dataset can be accessed on PhysioNet at [https://doi.org/10.13026/g02k-a047]. The Epilepsy Dataset is available on PhysioNet at [https://doi.org/10.13026/C2K01R]. The Alzheimer Dataset is stored on OpenNeuro with the persistent web link [10.18112/openneuro.ds004504.v1.0.4]. Lastly, the Bird et al. Dataset is hosted on Kaggle, and the data can be accessed at [https://www.kaggle.com/datasets/birdy654/eeg-brainwave-dataset-mental-state] The data collected from the survey conducted for this study are not publicly available due to privacy restrictions. However, the survey link can be provided upon request for interested individuals to participate in the survey. The findings and analysis derived from the survey data will be presented and discussed in this paper without disclosing any personally identifiable information. For more information or to request access to the survey link, please contact niam27832@hbku.edu.qa.
